# Association of rs3750920 polymorphism in *TOLLIP* with clinical characteristics of fibrosing interstitial lung diseases in Japanese

**DOI:** 10.1038/s41598-021-95869-9

**Published:** 2021-08-10

**Authors:** Takuma Isshiki, Kazuya Koyama, Sakae Homma, Susumu Sakamoto, Akira Yamasaki, Hiroshige Shimizu, Shion Miyoshi, Yasuhiko Nakamura, Kazuma Kishi

**Affiliations:** 1 Department of Respiratory Medicine, Toho University Omori Medical Center, Ota-ku Omori nisi 6-11-1, Tokyo, 143-8541 Japan; 2 Department of Advanced and Integrated Interstitial Lung Diseases Research, School of Medicine, Toho University, Tokyo, Japan; 3 Department of Respiratory Medicine and Rheumatology, Graduate School of Biomedical Sciences, Tokushima University, Tokushima, Japan

**Keywords:** Respiratory tract diseases, Medical research

## Abstract

*TOLLIP* polymorphism has been implicated in the development and prognosis of idiopathic pulmonary fibrosis (IPF), mainly in whites. However, ethnic differences in the characteristics of other interstitial pneumonia (non-IPF) subtypes are unclear. We evaluated the association between the rs3750920 genotype and the clinical characteristics of Japanese patients with fibrosing interstitial lung diseases (ILD). We genotyped 102 patients with fibrosing ILD (75 IPF and 27 non-IPF patients) and analyzed the interaction between the rs3750920 genotype distribution and their clinical characteristics. The overall frequencies of the C/C, C/T, and T/T genotypes were 69%, 25%, and 6%, respectively. The proportion of minor T allele carriers was larger in IPF patients than in non-IPF patients (37% vs. 15%, P = 0.031). In addition, survival at 3 years was significantly better for carriers than for non-carriers of the T allele. There was no significant association between genotype distribution and change in pulmonary function after introduction of antifibrotic agents. The frequency of the minor T allele of rs3750920 was low in Japanese patients with fibrosing ILD, particularly in non-IPF patients. Carriers of the minor T allele had better survival than non-carriers. Presence of the T allele might thus be an indicator of better outcomes for fibrosing ILD.

## Introduction

Idiopathic pulmonary fibrosis (IPF) is a lung disorder characterized by fibrosis of the lung interstitium. Although IPF is the most common and most severe subtype of chronic fibrosing interstitial pneumonia^1^, other interstitial lung diseases (ILD) such as non-specific interstitial pneumonia (NSIP), connective tissue disease-associated ILD (CTD-ILD), fibrotic hypersensitivity pneumonitis (HP), unclassifiable ILD (UCIP), and pleuroparenchymal fibroelastosis also have a progressive fibrotic phenotype. IPF is distinct from non-IPF ILD; however, IPF and non-IPF ILD share some morphological features and pathological mechanisms^2^. Findings suggesting a common fibrotic pathogenesis in pulmonary fibrosis have led to recent clinical trials of antifibrotic agents that target not only IPF but also other types of fibrosing ILD^3,4^, and to the coining of the term progressive-fibrosing interstitial lung diseases (PF-ILD)^3,5^.

Among host factors for ILD, genomic factors have been implicated as risk factors for developing pulmonary fibrosis and were reported to be associated with clinical outcomes. Patients with telomere-related rare variants in *TERT*, *TERC*, *PANR*, and *RTEL1* had a higher risk of pulmonary fibrosis development and progression and worse outcomes for a variety ILD types^6^. In addition, shortened leukocyte telomere length was related to poor outcomes for IPF and HP^7–9^. In addition to these findings regarding telomere length and telomere-maintenance genes, a genome-wide association study (GWAS) found that single nucleotide polymorphisms (SNPs) in *MUC5B* and *TOLLIP* might be associated with IPF susceptibility and clinical outcome^10,11^. Interestingly, the rs3750290 genotype—a functional SNP within *TOLLIP*—interacted with IPF outcome in post hoc analysis of the PANTHER trial: a randomized clinical trial of prednisone, azathioprine, and *N*-acetylcysteine (NAC) for IPF^12,13^. In that study, the T/T genotype of rs3750920 was associated with significantly better survival after NAC therapy, as compared with placebo, while patients with the C/C genotype had worse survival after NAC treatment^13^. Thus, there may be drug–gene interaction, and genotype-stratified use of antifibrotic agents should be considered in IPF treatment. However, the association of rs3750920 polymorphism with other types of fibrosing ILD was not analyzed. In addition, the current guidelines for IPF^1^ recommend nintedanib and pirfenidone as the main treatment for fibrosis; NAC treatment is not the current standard. Thus, associations with other antifibrotic agents should be analyzed. Moreover, ethnic differences may exist; however, the present genomic data for IPF have mostly been collected from white populations, and it is unclear if these data are applicable to non-white populations, including Asian populations.

This study examined associations of *TOLLIP* rs3750290 SNPs with the clinical characteristics of Japanese patients with fibrosing ILD.

## Methods

### Patients

We recruited and collected blood samples from 102 patients who were treated for fibrosing ILD at our institution during the period from 2018 through 2020. The following patient characteristics were extracted from medical records: age, sex, smoking history, laboratory data, pulmonary function test results, gender-age-physiology score, development of acute exacerbation, treatment for fibrosing ILD, and outcome. IPF and other fibrosing ILD were diagnosed by using the guidelines of the American Thoracic Society (ATS)/European Respiratory Society (ERS)/Japanese Respiratory Society/Latin American Thoracic Association and the statement of the ATS/ERS^1,5^. Acute exacerbation of fibrosing ILD was diagnosed by using criteria reported by the international working group on acute exacerbation of IPF^14^. The data were locked at the end of 2020. The institutional review board of Toho University Graduate School of Medicine approved this study (A18043), and the research was performed in accordance with relevant guidelines/regulation. All patients provided written informed consent.

### DNA extraction and genotyping

Peripheral whole-blood samples were obtained from patients and stored at − 80 °C. Genomic DNA was extracted with a High Pure PCR Template Preparation Kit (Roche Diagnostics, Mannheim, Germany), in accordance with the manufacturer’s instructions. The genotype of rs3750290 was determined by real-time PCR using a TaqMan SNP Genotyping Assay (Thermo Fisher Scientific Japan, Tokyo, Japan). Real-time PCR was conducted with an Applied Biosystems QuantStudio 3D Real-time PCR system (Thermo Fisher Scientific Japan, Tokyo, Japan).

### Data analysis

Continuous variables were analyzed with the unpaired t-test when comparing two groups and with one-way ANOVA when comparing more than two groups, as appropriate. Categorical variables were compared with the χ^2^ test and Fisher exact test. We used the Kaplan–Meier method to analyze outcomes. The log-rank test was used to compare two groups. Cox proportional hazards analysis was used to identify independent predictors of survival. All P values are two-sided, and a *P* value of less than 0.05 was considered to indicate statistical significance. Statistical analysis was done by using SPSS version 27 (SPSS Inc., IL, USA) and PRISM version 8 (MDF Co., Ltd., CA, USA).

## Results

### Baseline characteristics of patients and rs3750920 genotype distribution

The baseline characteristics of the 102 patients with fibrosing ILD are shown in Table 1. Among these patients, 74% had IPF. Among those with CTD-ILD, 3 had systemic sclerosis, 2 had rheumatoid arthritis, and 1 had mixed connective tissue disease. All UCIP patients (n = 3) underwent surgical lung biopsy and had pathological findings indicating combined usual interstitial pneumonia and NSIP.

**Table 1:** Baseline characteristics of patients.

	All patients
No	102
Age, years	73 ± 7
Sex, male, n	76 (75%)
Smoking history, yes, n	71 (70%)
**Clinical diagnosis of fibrosing ILD, n**	
IPF	75 (74%)
NSIP	10 (10%)
CTD-ILD	6 (6%)
HP	2 (2%)
UCIP	3 (3%)
PPFE	6 (6%)
**Laboratory data**	
LDH, IU/L	251 ± 60
SP-D, ng/dL	277 ± 227
KL-6, U/mL	1091 ± 799
**Pulmonary function testing**	
FVC, mL	2356 ± 882
%FVC, %	77.9 ± 24.9
FEV1%, %	80.0 ± 18.1
%DLco, %	64.8 ± 22.5

Data are presented as mean ± SD.

*ILD* interstitial lung diseases, *IPF* idiopathic pulmonary fibrosis, *NSIP* non-specific interstitial pneumonia, *CTD-ILD* connective tissue disease–associated interstitial lung disease, *HP* fibrotic hypersensitivity pneumonitis, *UCIP* unclassifiable interstitial lung disease, *PPFE* pleuroparenchymal fibroelastosis, *LDH* lactate dehydrogenase, *SP-D* surfactant protein-D, *KL-6* Krebs von den Lungen-6, *FVC* forced vital capacity, *FEV* forced expiratory volume in 1 s, *DLco* diffusing capacity for carbon monoxide.

The genotype distribution of rs3750920 in relation to fibrosing ILD subtype is shown in Table 2. DNA extraction and rs3750920 genotyping was successful in all patients. Among the 102 patients, 70 (69%), 26 (25%), and 6 (6%) had the C/C, C/T, and T/T genotypes, respectively. Only 1 of the 27 non-IPF patients had the T/T genotype. The frequency of rs3750920 minor T allele carriers was higher in IPF patients than in non-IPF patients (37% vs. 15%, P = 0.031).

**Table 2:** Genetic and allele distribution of rs3750920.

	Genotype			Allele	
	C/C	C/T	T/T	C	T
Total (n = 102)	70 (69%)	26 (25%)	6 (6%)	82%	18%
HWE	66%	30%	4%		
IPF (n = 75)	47 (63%)	23 (30%)	5 (7%)	78%	22%
HWE	61%	34%	5%		
Non-IPF (n = 27)	23 (85%)	3 (11%)	1 (4%)	91%	9%
HWE	82%	17%	1%		
NSIP (n = 10)	8 (80%)	1 (10%)	1 (10%)	85%	15%
CTD-ILD (n = 6)	5 (83%)	1 (17%)	0 (0%)	92%	8%
HP (n = 2)	2 (100%)	0 (0%)	0 (0%)	100%	0%
UCIP (n = 3)	2 (67%)	1 (33%)	0 (0%)	84%	16%
PPFE (n = 6)	6 (100%)	0 (0%)	0 (0%)	100%	0%

*IPF* idiopathic pulmonary fibrosis, *NSIP* non-specific interstitial pneumonia, *CTD-ILD* connective tissue disease-associated interstitial lung disease, *HP* fibrotic hypersensitivity pneumonitis, *UCIP* unclassifiable interstitial lung disease, *PPFE* pleuroparenchymal fibroelastosis, *HWE* expected frequency calculated by using the Hardy–Weinberg equilibrium.

### Characteristics of IPF patients in relation to rs3750920 genotype

Table 3 shows the characteristics of IPF patients (n = 75) in relation to genotype. At baseline there was no significant difference among the 3 groups in sex, age, severity score, pulmonary function, or serological markers. In addition, the incidence of acute exacerbation, lung cancer complication rate, and IPF treatment were similar for the three groups during the observation period.

**Table 3:** Clinical characteristics of IPF patients, by rs3750290 genotype.

	C/C	T/C	T/T
No	47	23	5
Age, years	73 ± 8	68 ± 9	71 ± 11
Sex, male, n	39 (83%)	20 (87%)	4 (80%)
Smoking history, yes, n	37 (79%)	18 (78%)	4 (80%)
GAP stage (1/2/3/NA)	25/19/1/0	17/4/1/1	3/2/0/0
**Laboratory data**			
LDH, IU/L	262 ± 60	245 ± 45	244 ± 47
SP-D, ng/dL	294 ± 227	219 ± 155	349 ± 419
KL-6, U/mL	1052 ± 689	1079 ± 626	1678 ± 850
**Pulmonary function testing**			
FVC, mL	2539 ± 813	2700 ± 604	2592 ± 922
%FVC, %	81.9 ± 21.5	85.6 ± 14.4	82.5 ± 16.8
FEV1%, %	82.4 ± 9.0	82.8 ± 6.7	83.8 ± 7.0
%DLco, %	68.8 ± 21.5	68.5 ± 15.3	54.5 ± 12.0
AE development, yes, n	13 (28%)	7 (30%)	2 (40%)
Complications of lung cancer	3 (6%)	3 (13%)	1 (20%)
**Treatment for IPF**			
Prednisolone	16 (34%)	7 (30%)	4 (80%)
Pirfenidone	19 (40%)	11 (48%)	2 (40%)
Nintedanib	20 (43%)	12 (52%)	3 (60%)
Inhaled NAC	2 (4%)	2 (9%)	1 (20%)

Data are presented as mean ± SD.

*IPF* idiopathic pulmonary fibrosis, *NA* not available, *GAP* gender-age-physiology, *LDH* lactate dehydrogenase, *SP-D* surfactant protein-D, *KL-6* Krebs von den Lungen-6, *FVC* forced vital capacity, *FEV* forced expiratory volume in 1 s, *DLco* diffusing capacity for carbon monoxide, *AE* acute exacerbation of idiopathic pulmonary fibrosis, *ILD* interstitial lung diseases, *NAC* *N*-acetylcysteine.

### Association between outcome and rs3750920 genotype

**Figure 1:**
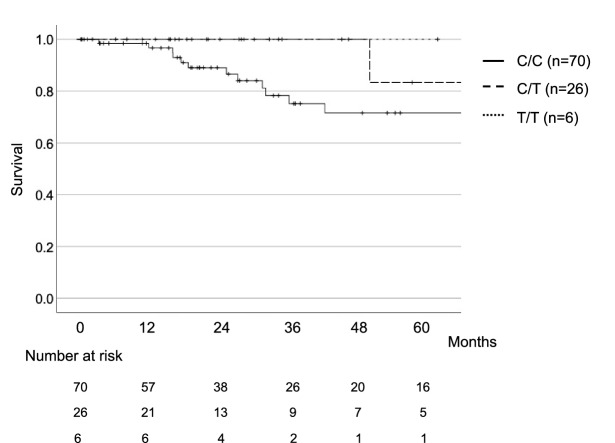
Kaplan–Meier survival curves for each rs3750920 genotype (n = 102).

Kaplan–Meier curves were used to analyze outcome in relation to genotype. Figure 1 shows the survival curve for each genotype from the first visit to our institution. There was no significant difference in outcome between the three groups.

**Figure 2:**
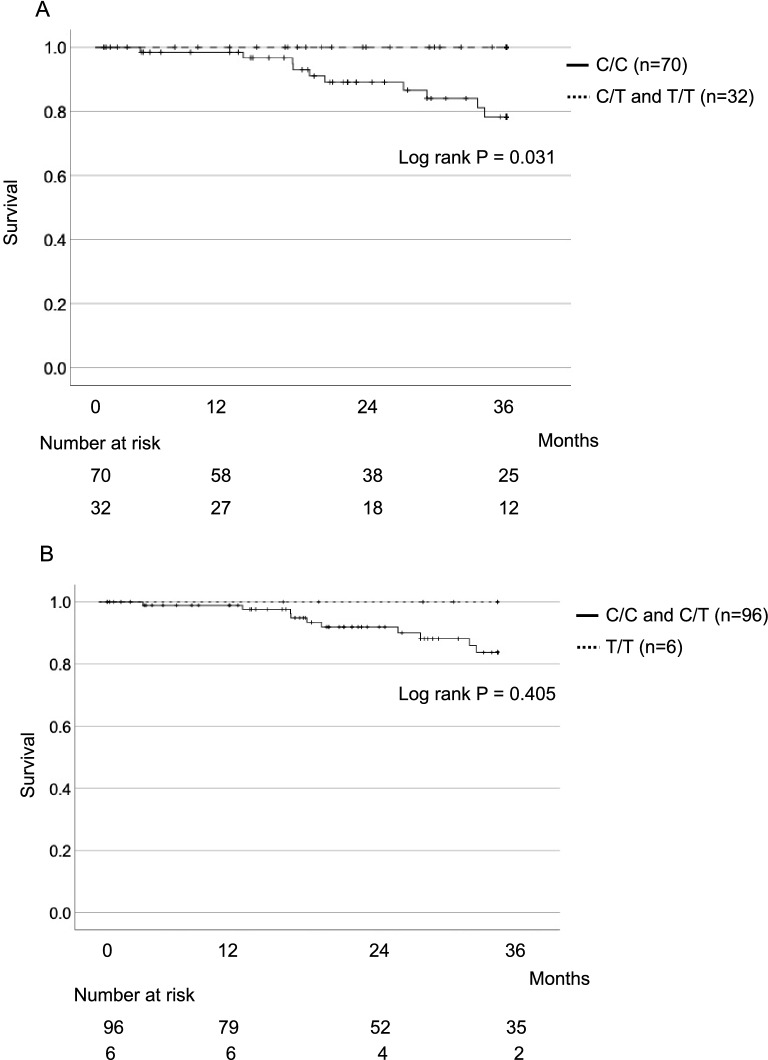
**(A)** Kaplan–Meier survival curves for carriers (n = 32) and non-carriers (n = 70) of the rs3750920 T allele. Carriers had significantly better survival at 3 years (P = 0.031). **(B)** Kaplan–Meier survival curves for carriers (n = 96) and non-carriers (n = 6) of the rs3750920 C allele.

**Figure 3:**
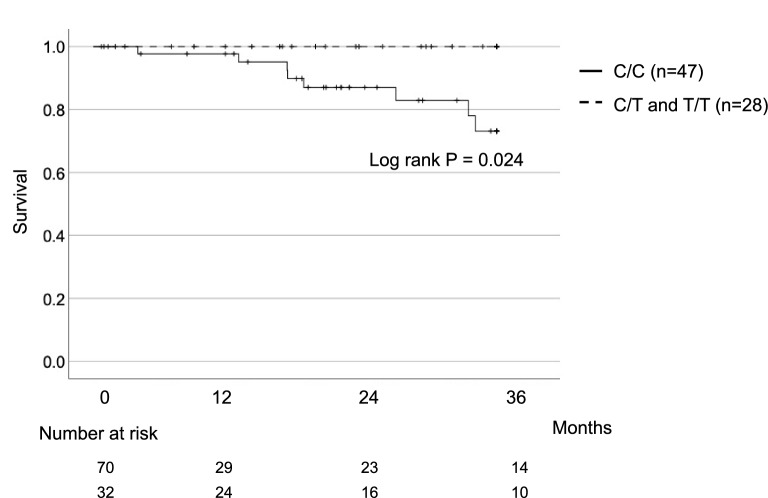
Kaplan–Meier survival curves for idiopathic pulmonary fibrosis (IPF) patients identified as carriers (n = 28) and non-carriers (n = 47) of the rs3750920 T allele. Carriers had significantly better survival at 3 years (P = 0.024).

Next, we directly compared outcomes for fibrosing ILD in carriers and non-carriers of the T allele of rs3750920. As shown in Fig. 2A, three-year survival was significantly better for carriers than for non-carriers of the T allele (100% vs 78%, P = 0.031). Although there was no significant difference in survival in the recessive model (Fig. 2B), when non-IPF patients were excluded from the analysis, the Kaplan–Meier curves were similar to those in Fig. 2A: 3-year survival remained significantly better for T allele carriers than for non-carriers (100% vs 73%, P = 0.024) (Fig. 3). However, the minor T allele was not a significant independent predictor in Cox proportional multivariate analysis when examined with percent forced vital capacity and use of antifibrotic treatment (data not shown).

### Change in pulmonary function after introduction of antifibrotic agents, by genotype

**Figure 4:**
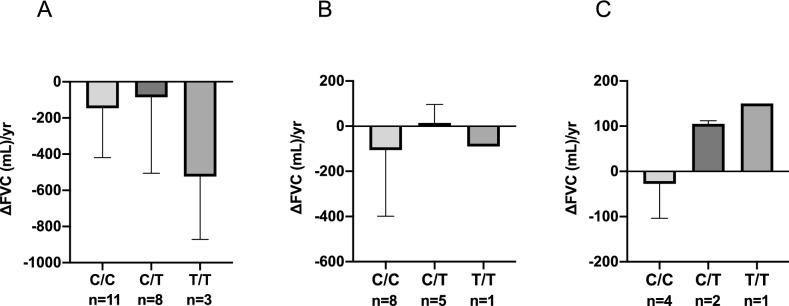
Change in forced vital capacity (FVC) from baseline to 1 year after the start of antifibrotic therapy, by rs3750920 genotype. **(A)** Patients treated with nintedanib (n = 22). **(B)** Patients treated with pirfenidone (n = 14). **(C)** Patients treated with inhaled *N*-acetylcysteine (n = 7).

Finally, we analyzed the pulmonary function of patients who received antifibrotic agents, including inhaled NAC, during the observation period. Change in FVC during the interval from the start of an antifibrotic agent to 1 year later was compared. We were only able to analyze 22 patients treated with nintedanib (Fig. 4A), 14 patients treated with pirfenidone (Fig. 4B), and 7 patients treated with inhaled NAC (Fig. 4C). Change in FVC did not differ in relation to rs3750290 genotype for any antifibrotic agent.

## Discussion

We observed an association between the rs3750290 genotype in *TOLLIP* and the clinical characteristics of Japanese patients with fibrosing ILD*.* The frequencies of the C/C, C/T, and T/T genotypes were 69%, 25%, and 6%, respectively, in the total sample and 63%, 30%, and 7% in IPF patients. The present findings and the respective genotype distribution of 22%, 50%, and 28% for patients in the PANTHER trial^13^ suggest that Japanese are less likely than whites to be carriers of the rs3750920 minor T allele. Similarly, the genotype distribution of SNPs in *MUC5B* (rs35705950) were different in Japanese IPF and NSIP patients than in a German cohort^15^. The rs35705950 minor T allele was present in 3.4% of IPF patients and 1.7% of NSIP patients in Japanese cohorts, as compared with 33.1% and 27.4%, respectively, in German cohorts. Thus, the minor T allele might be less frequent in *MUC5B* (rs35705950) and *TOLLIP* (rs3750920) SNPs in Japanese. SNP data provided by the National Center for Biotechnology Information showed a rs3750920 T allele frequency of 43% in Europeans and 32% in east Asians^16^. A Japanese genetic database (TOGO VAR) indicated that 23% of Japanese had the minor T allele^17^. The lower frequency of the minor T allele in Japanese subjects compared to European subjects might explain why the proportion of T allele carrier is lower in our IPF patients than in IPF patients in the PANTHER cohort. However, the frequency of the T allele was higher in our patients, especially IPF patients (37%), than in subjects of Japanese database. These findings suggest that there may be an association between IPF development and rs3750920 polymorphism, as indicated by previous GWAS^10,11^.

The proportion of T allele carriers was significantly higher in IPF patients than in non-IPF patients in our study. To our knowledge, this is the first report to compare genotype distribution between IPF and other fibrosing ILD subtypes. Oldham et al. reported that the TT genotype of rs3750920 was more frequent in antinuclear antibody (ANA)–positive (ANA titer ≥ 1:320) patients with IPF and interstitial pneumonia with autoimmune features than in those who were ANA-negative^18^. Although we could not assess genetic distribution in relation to ANA seropositivity in our patients, this tendency was not observed in our patients with CTD-ILD, NSIP, and UCIP.

Previous studies of the association between rs3750920 genotype and outcome have yielded ambiguous results. In post hoc analysis in the PANTHER trial, survival in IPF patients treated with NAC differed according to rs3750920 genotype: T/T was associated with better outcomes, and outcomes were worse for patients with C/C than for the placebo treatment group^13^. In contrast, a recent study of 62 white patients with IPF reported no association of survival with rs3750920 genotype^19^. The present patients with the T allele had significantly better 3-year survival than did those without the T allele, regardless of treatment. This difference remained even when we limited the analysis to IPF patients. However, since the prevalence of the minor T allele was low, especially in non-IPF patients (only 3 patients had C/T and 1 patient had T/T), it was difficult to conduct survival analysis of non-IPF patients. Moreover, because we could not validate the association of the minor T allele of rs3750920 with better outcomes for fibrosing ILD in multivariate analysis, we cannot conclude that rs3750920 genotype is associated with the outcome for fibrosing ILD. We also analyzed the association between decline in FVC and treatment with the antifibrotic agents pirfenidone, nintedanib, and NAC, for each genotype. NAC has been used as inhaled form in Japan^20,21^. Although we found no significant interaction between rs3750920 genotype and antifibrotic treatment in our small sample, it is intriguing that FVC improved after inhaled NAC therapy in all 3 patients with the T allele (Fig. 4). A future study with a larger cohort will be required in order to validate how rs3750920 genotype is related to outcome and responsiveness to treatment with antifibrotic agents.

The biological mechanisms underlying the interaction of *TOLLIP* polymorphism with the pathogenesis of pulmonary fibrosis are not fully understood. Toll-like receptors (TLRs) play a pivotal role in the innate immune system and inflammatory response^22^. Activation of TLRs by exogenous pathogen–associated molecular patterns triggers inflammatory signaling pathways, which results in suppression of infection. In addition, TLRs recognize host-derived endogenous ligands such as pathogen-associated molecular patterns and are involved in regulating non-infectious tissue injury^23^. TLR2 and TLR4 are localized on the surface of cells such as alveolar macrophages and lung epithelial cells in the lungs and are crucial in regulating inflammatory response and fibrosis^24^. *TOLLIP* encodes toll-interacting protein (TOLLIP), which is a negative regulator of TLRs, including TLR2 and TLR4^25^. TOLLIP is an intracellular adaptor molecule that can bind to IL-1R–associated kinases (IRAK-1) and inhibit activation of nuclear factor kappa B (NFκB), which results in resolution of the inflammatory response caused by the TLRs/ligand signal pathway^26^. rs3750920 is a functional synonymous variant coding SNPs in *TOLLIP* exon 3; it was marginally associated with IPF susceptibility in 2 GWAS studies^10,11^. TOLLIP mRNA levels were significantly higher in persons with the T/T rs3750920 genotype than in those with the C/C and C/T genotypes in human samples^25^. Thus, increased TOLLIP expression by the minor allele might be associated with pathogenesis, responsiveness to treatment, and/or ILD prognosis. However, the complexity of immune homeostasis complicates our understanding of pathogenesis. TLR2 signaling was reported to promote pulmonary fibrosis in a model of bleomycin-induced pulmonary fibrosis^27,28^. Inhibition of TLR4 exacerbated bleomycin-induced pulmonary fibrosis^29^, while another study reported that TLR4 enhanced fibroblast activity, thereby promoting wound healing and fibrosis^30^. In addition, TOLLIP inhibited TLR4 downstream and suppressed inflammation under acute inflammatory conditions. In contrast, in low-grade, chronic inflammation, TOLLIP translocated to mitochondria and facilitated chronic inflammation^26^. The link between SNPs and the pathogenesis of pulmonary fibrosis should be further investigated.

This study has several limitations. First, it was conducted at a single center and the sample size was small. In addition, we noted no significant difference in overall survival, perhaps because the observational period was short (108 ± 104 months). A nationwide Japanese analysis of genomic factors in idiopathic interstitial pneumonia is ongoing (NEJ036A: UMIN000032117) and might validate our findings. Second, we could not directly compare the genetic distribution of rs3750920 in our patients and healthy Japanese subjects. However, international and Japanese SNP databases provided useful information for analysis of the association of genetic distribution and disease.

The present findings indicate that few Japanese with fibrosing ILD, especially those without IPF, are carriers of the rs3750920 minor T allele. Survival might be better for carriers than for non-carriers of the T allele. However, genotype was not associated with change in pulmonary function after treatment with any antifibrotic drug. To our knowledge, this is the first study to investigate associations of clinical characteristics, including outcome for fibrosing ILD, with rs3750920 genotype in a non-white population. Despite the limitations of this study, our results should provide insights regarding IPF and fibrosing ILD treatment. A nationwide Japanese genomic study is ongoing and might validate the present findings.
